# Factors Affecting the Adoption of Electronic Prescription by Physicians and Pharmacists

**DOI:** 10.1002/hsr2.70805

**Published:** 2025-05-05

**Authors:** Mahdie ShojaeiBaghini

**Affiliations:** ^1^ Student Research Committee, Faculty of Management and Medical Information Sciences Kerman University of Medical Sciences Kerman Iran

**Keywords:** electronic prescribing, e‐prescribing, pharmacists, physicians, technology acceptance

## Abstract

**Background and Aims:**

Electronic prescribing holds significant potential for improving healthcare quality and efficiency. However, its success relies on healthcare professionals' acceptance and use. This study investigated the factors influencing e‐prescribing adoption among physicians and pharmacists in Iran, where its use has been mandated since December 2021.

**Methods:**

A cross‐sectional study was conducted in 2024 using a structured questionnaire to gather data from 189 physicians and pharmacists in Kerman, Iran. The data collection tool was a structured questionnaire divided into two sections: demographic information and research variables. The questionnaire measured key constructs based on the Technology Acceptance Model (TAM) and the Unified Theory of Acceptance and Use of Technology (UTAUT). Data were analyzed using SPSS 25.0 and MPLUS 8.3.2 software.

**Results:**

A total of 171 individuals participated in our survey. The updated measurement model confirmed the presence of convergent and discriminant validity, as well as reliability and fit. Analysis of the structural model indicated that, at the 95% confidence level, perceived ease of use positively influences perceived usefulness, which in turn enhances trust in the e‐prescribing system. Additionally, technology self‐efficacy has a positive impact on attitudes toward using e‐prescribing.

**Conclusions:**

This study presents valuable insights into the factors influencing the adoption of e‐prescribing within the Iranian healthcare context. The findings carry significant implications for policymakers, system developers, and healthcare administrators aiming to enhance the implementation of e‐prescribing and promote its widespread adoption. Furthermore, this study contributes to the broader discourse on health information technology adoption by emphasizing the critical importance of contextual factors in technology acceptance research.

## 1 Introduction

In today's healthcare landscape, the integration of health information technology (HIT) is essential for enhancing care quality, safety, and efficiency [1, 2, 3, 4, 5]. This encompasses a broad spectrum of applications, from electronic health records and telemedicine to clinical decision support systems and electronic prescribing (e‐prescribing) [6, 7]. E‐prescribing, in particular, has garnered significant attention due to its potential to mitigate the challenges associated with traditional prescription methods, such as medication errors, illegibility, and fraud [8, 9, 10]. By streamlining the communication between physicians and pharmacies, e‐prescribing not only improves workflow but also promises substantial healthcare cost savings [11, 12, 13]. Nonetheless, the success of e‐prescribing hinges on its acceptance by healthcare professionals and the broader medical community [14, 15, 16, 17].

Numerous theories and models have been developed to understand technology acceptance, including the Technology Acceptance Model (TAM), Theory of Planned Behavior (TPB), Unified Theory of Acceptance and Use of Technology (UTAUT), Task‐Technology Fit (TTF) model, and the Technology–Organization–Environment (TOE) framework [18, 19, 20, 21, 22]. These frameworks offer valuable insights into the interplay between technology and organizational dynamics [14]. While each model contributes uniquely, relying on just one can be limiting. Therefore, an integrated approach is essential for a comprehensive understanding of Health Information Technology (HIT) acceptance, especially in the context of e‐prescribing. By combining different frameworks, we can enhance the predictability of acceptance indicators and ensure that research is cost‐effective [14, 23, 24, 25]. This holistic approach is crucial for examining technology management across diverse technologies, services, and demographics [14, 23, 25, 26].

Despite this recognized need, research on e‐prescribing acceptance often relies on single models or theories [15, 27, 28, 29, 30], creating a significant gap, especially concerning physician adoption. This gap is particularly pronounced in regions like Iran, where e‐prescribing adoption remains underexplored. Iran's healthcare system faces distinct challenges in implementing e‐prescribing. These challenges include limited infrastructure in underprivileged areas, varying levels of digital literacy among healthcare professionals, specific regulatory hurdles, poor management practices, cultural attitudes towards technology, and the presence of multiple insurance systems [31, 32, 33]. Considering the contextual and cultural influences on technology acceptance [23, 34], research tailored to the specific challenges and opportunities within the Iranian healthcare system is crucial. This study aims to address this gap by investigating the factors influencing e‐prescribing acceptance among Iranian healthcare providers. By utilizing an integrated theoretical framework, this study seeks to offer valuable insights into the adoption and implementation of HIT. Ultimately, it aims to enhance the understanding of e‐prescribing in the Iranian context.

This article is presented in compliance with the STROBE report checklist.

## 2 Methods

### 2.1 Study Design

This study was conducted as a cross‐sectional study in 2024, targeting physicians and pharmacists who utilize electronic prescription systems. Due to time constraints, the nature of cross‐sectional data collection, budget considerations, and other factors, the study focused specifically on professionals based in Kerman. Kerman was selected as the study site because it is the center of the largest province in Iran and attracts numerous patients from neighboring provinces to its medical facilities. Participants were selected through a simple random sampling technique. Questionnaires served as the primary tool for data collection. To achieve the established sample size, researchers personally visited clinics, pharmacies, and other locations specified by respondents to administer the questionnaires.

### 2.2 Data Sample

The total number of physicians and pharmacists practising in Kerman is estimated to be approximately 7000. Considering the structural equation methodology employed in this study, the sample size was determined using GPower software version 3.1, yielding a target of 189 participants. This calculation was based on a significance level (α) of 0.01, a statistical power of 0.95, an effect size of 0.15, and the inclusion of six predictors. To mitigate potential non‐cooperation and reduce sample dropout, the number of distributed questionnaires was increased by 10%. Participants were selected through a simple random sampling method. Field interviewers conducted personal visits to hospitals and pharmacies in Kerman to distribute questionnaires to eligible physicians and pharmacists. Eligibility criteria included prior experience with electronic prescription systems and a willingness to participate in the study. This focused approach resulted in a notable response rate of 86%, highlighting the effectiveness of direct, face‐to‐face survey administration.

### 2.3 Data Collection

The data collection instrument utilized in this study was a structured questionnaire consisting of two distinct sections. The first section focused on collecting essential demographic information, including gender, age, work experience with electronic prescribing systems, and levels of expertise. The second section comprised 34 carefully designed items aimed at evaluating the study's key variables: technology self‐efficacy (three items) [35], perceived ease of use (six items) [36], perceived usefulness (six items) [37], trust (five items) [38], peer influence (five items) [39], attitude toward use (six items) [40], and actual use (three items) [41]. These variables were identified through a comprehensive review of the literature on technology acceptance, as they are critical factors that significantly influence user adoption of health information technology systems. The questionnaire items were developed using established scales from prior research and then translated into Persian. Five experts in health information technology, as well as experienced physicians and pharmacists, reviewed the translations to ensure the validity and clarity of the content. Then, the items were refined based on their feedback. Notably, all variables were formative. Responses were recorded on a 5‐point Likert scale, ranging from “strongly agree” to “strongly disagree”.

### 2.4 Data Analysis

The data underwent a comprehensive initial screening and preprocessing to address issues such as unresponsive and anomalous responses, data gaps, and irregular distributions, thereby ensuring a robust sample size. Following this, SPSS 25.0 was utilized to generate descriptive statistics. To evaluate the proposed causal hypotheses within a structural equation modeling (SEM) framework, the study employed MPLUS version 8.3.2, facilitating a detailed analysis of the causal relationships between variables. The MPLUS path models are composed of two sets of linear equations: the measurement (or outer) model and the structural (or inner) model. The measurement model delineates the relationships between observed variables and latent constructs. Initially, the credibility was measured using Cronbach's alpha. Also, the convergent validity of the structural model was measured using the average variance extracted (AVE) and composite reliability (CR) tests, and the discriminant validity of the structural model was measured using the Fornell and Larcker test. Then, the fit of the structural model was examined using the Confirmatory Fit Index (CFI), Root Mean Square Error of Approximation (RMSEA), and Standardized Root Mean Square (SRMR) [42].

### 2.5 Ethical Approval

This study diligently adhered to ethical guidelines, ensuring that all participants provided verbal informed consent before their involvement. Participation was entirely voluntary, with participants receiving comprehensive information about the study's objectives and procedures. Confidentiality of participant data was maintained, and the highest standards of privacy were observed. Ethical clearance was obtained from the Research Ethics Committee of Kerman University of Medical Sciences in Kerman, Iran, under approval number IR.KMU.REC.1401.504.

## 3 Results

Following the initial screening, 171 out of 180 questionnaires were validated for analysis. The demographic profile showed that most respondents were male (57.9%), with the largest age group being those aged 40–50 years (29.8%). A significant portion of the participants were specialists (43.8%). Furthermore, 80.1% reported having experience with electronic prescription systems for a duration of 2–6 months, indicating their involvement with modern healthcare technology (Table 1).

**Table 1:** Basic characteristic of participants.

Variables	Categories	Frequency	Percentage (%)
Gender	Female	72	42.1
	Male	99	57.9
Age	< 30	27	15.8
	30–40	41	24.0
	40–50	51	29.8
	50–60	37	21.6
	> 60	15	8.8
Experience (month)	< 1	17	9.9
	2–6	137	80.1
	6–12	15	8.8
	12 >	2	1.2
Expertise	Specialist	75	43.8
	General practitioner	36	21.1
	Pharmacist	60	35.1

Peer influence exhibited the highest mean score (3.79), while actual use had the lowest mean score (1.30). Table 2 presents the descriptive statistics for each variable.

**Table 2:** Descriptive statistics for study variables.

Variables	Mean	Standard deviations	Ranges
Trust	3.62	0.979	1–5
Perceived ease of use	3.41	0.739	1–5
Perceived usefulness	3.58	0.625	1–5
Peer influence	3.79	0.433	1–5
Technology self‐efficacy	3.64	0.804	1–5
Attitude toward use	2.62	0.882	1–5
Actual use	1.30	0.408	1–5

### 3.1 Assessment of the Measurement Model

The initial confirmatory factor analysis (CFA) indicated that the original research model did not meet the required criteria for construct validity and reliability. Consequently, the model was refined based on software recommendations and insights from the literature. Subsequent evaluation confirmed that all variables had significant factor loadings, each exceeding the 0.5 threshold, thus supporting the integrity of the constructs.

All variables achieved Cronbach's alpha values above the acceptable minimum of 0.7, indicating strong reliability. The average variance extracted (AVE) for each variable exceeded 0.5, and the composite reliability (CR) scores surpassed the corresponding AVE scores, demonstrating robust internal consistency. The Fornell and Larcker criterion confirmed discriminant validity, as the square root of the AVE for each variable was greater than its correlation with any other variable (Table 3).

**Table 3:** Convergent and discriminant validity indices.

Index	AVE	CR	Fornell and Larcker test						
			Actual use	Attitude toward use	Technology self‐efficacy	Peer influence	Perceived usefulness	Trust	Ease of use
Actual use	0.855	0.680	**0.825**						
Attitude toward use	0.885	0.575	−0.075	**0.758**					
Technology self‐efficacy	0.861	0.674	−0.277	0.28	**0.821**				
Peer influence	0.777	0.515	−0.164	0.002	0.271	**0.644**			
Perceived usefulness	0.897	0.596	−0.104	−0.085	−0.064	−0.156	**0.772**		
Trust	0.817	0.507	−0.054	−0.054	−0.092	−0.075	0.388	**0.691**	
Ease of use	0.856	0.514	−0.011	0.008	0.036	−0.134	0.71	0.432	**0.717**

Abbreviations: AVE, average variance extracted; CR, composite reliability.

Table 4 shows that the modified measurement model demonstrates excellent fit, meeting both theoretical and empirical standards. This strong alignment suggests that the sample observations effectively represent the broader target population.

**Table 4:** Fit indices of the modified measurement model.

Metric	Estimate	Threshold	Interpretation
Χ^2^	304.865	n/a	n/a
DF	149	n/a	n/a
CFI	0.928	> 0.900	pass
RMSEA	0.078 (0.066, 0.091)	< 0.100	pass
SRMR	0.050	< 0.080	pass

Abbreviations: CFI, confirmatory fit index; DF, degree of freedom; RMSEA, root mean square error of approximation; SRMR, standardized root mean square.

### 3.2 Assessment of the Structural Model

**Figure 1:**
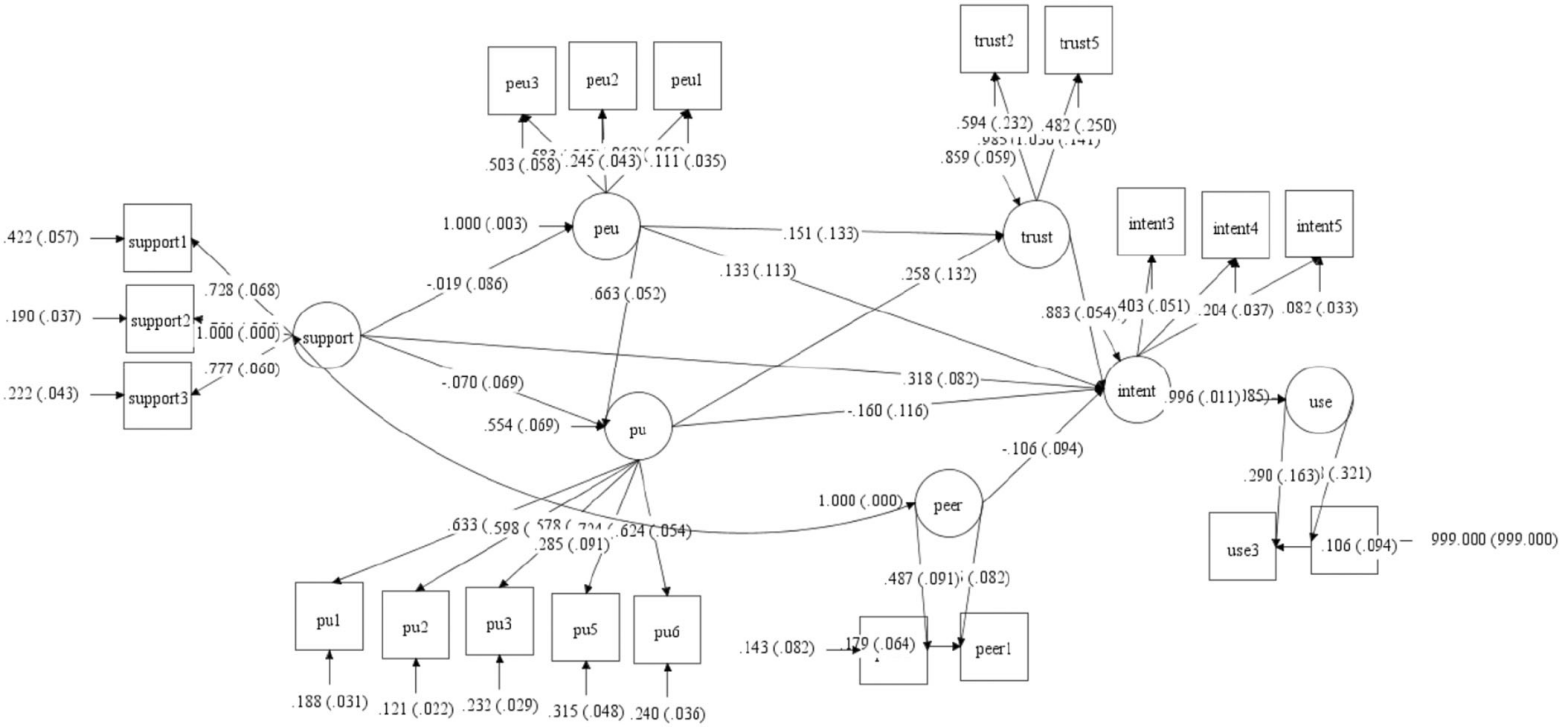
Structural model in standard estimation mode. *Note:* support, technology self‐efficacy; PU, perceived usefulness; PEU, perceived ease of use; trust, trust; peer, peer influence; intent, attitude toward use; use, actual use.

Having established reliability, validity, and the alignment of observations with the data, the analysis proceeded to examine the structural model. This model allows for the testing of hypotheses regarding the causal relationships between the constructs. Figure 1 illustrates the structural model in standard estimation mode, depicting the relationships between the independent and dependent variables. The figure demonstrates that perceived ease of use positively and significantly influences perceived usefulness, which in turn positively and significantly affects trust. Additionally, technology self‐efficacy shows a positive and significant impact on attitudes toward use.

Analysis of the structural model revealed that three of the 11 research hypotheses were supported at the 95% confidence level, indicating positive relationships between independent and dependent variables. Perceived ease of use had a positive and significant effect on perceived usefulness (*p* = 0.001), and perceived usefulness had a positive and significant effect on trust (*p* = 0.001). Technology self‐efficacy also had a positive and significant effect on attitude toward use (*p* = 0.050).

The standardized path coefficients (β) confirmed that the effects on the dependent variables were positive and in line with expectations. Table 5 shows the results of the structural equation modeling, including standardized path coefficients (β), *p*‐values, and *R*‐squared values for the relationships between the constructs. It demonstrates the strength and statistical significance of the hypothesized relationships.

**Table 5:** Path coefficients and hypothesis testing results.

Hypothesis	Path	β	*p* value	SE	EST/SE	Result
H1	Technology self‐efficacy ‐> perceived ease of use	−0.019	0.827	0.086	−0.219	Rejected
H2	Technology self‐efficacy ‐> perceived usefulness	−0.070	0.313	0.069	−1.009	Rejected
H3	Perceived ease of use ‐> perceived usefulness	0.663	0.001	0.052	12.769	Accepted
H4	Perceived usefulness ‐> trust	0.258	0.050	0.132	1.960	Accepted
H5	Perceived ease of use ‐> trust	0.151	0.255	0.133	1.138	Rejected
H6	Technology self‐efficacy ‐> attitude toward use	0.318	0.001	0.082	3.862	Accepted
H7	Perceived usefulness ‐> attitude toward use	−0.160	0.168	0.116	−1.379	Rejected
H8	Perceived ease of use ‐> attitude toward use	0.133	0.240	0.113	1.175	Rejected
H9	Trust ‐> attitude toward use	−0.032	0.732	0.095	−0.342	Rejected
H10	Peer influence ‐> attitude toward use	−0.106	0.259	0.094	−1.128	Rejected
H11	Attitude toward use ‐> actual use	−0.063	0.085	−0.736	0.462	Rejected

## 4 Discussion

Since December 2021, Iran has mandated the use of electronic prescription systems. The success of e‐prescribing depends on user acceptance, especially among physicians and pharmacists. This study examined 11 hypotheses related to the factors influencing this acceptance, but only three were supported by the findings. This discussion will focus on these key hypotheses, their implications, and potential reasons why the others were not supported.

This study did not find a significant effect of technology self‐efficacy on perceived ease of use or perceived usefulness, contradicting prior research [43, 44, 45, 46]. Venkatesh and Davis previously highlighted that computer self‐efficacy is essential in determining perceived ease of use, both before and after users interact with a system [46]. The differing results in this study may be due to several factors, particularly the mandatory use of e‐prescribing. In Iran, the requirement to utilize e‐prescribing may have reduced the influence of self‐efficacy. The mandatory nature of the e‐prescribing system could potentially diminish the significance of self‐efficacy in this context. Poor user experience with an electronic prescribing system can lead to the rejection of hypotheses regarding the ease of use and usefulness of the system. If physicians encounter a complex user interface or numerous technical problems, they may find the system inefficient and challenging to use, resulting in negative attitudes toward it.

According to expectations and based on studies, perceived ease of use had a positive and significant effect on perceived usefulness. When users find a system intuitive and straightforward, they are more likely to recognize its value. This means that perceived ease can lead to greater perceived usefulness. This relationship is supported by numerous studies that highlight the crucial role of perceived ease of use in shaping perceptions of usefulness [47, 48]. Therefore, promoting ease of use can lead to greater acceptance and success of such technologies in healthcare settings [49].

As anticipated, perceived usefulness has a significant and positive impact on trust. Conversely, perceived ease of use does not significantly influence trust levels. The concepts of perceived ease of use and perceived usefulness underscore that individuals are more inclined to adopt technology they deem valuable and user‐friendly [16]. Several previous studies have illustrated that trust is closely associated with both perceived usefulness and ease of use [50, 51]. This highlights the importance of emphasizing a technology's utility to build trust among users. Users may be compelled to utilize the system irrespective of their confidence levels, potentially resulting in a disconnection between trust and the perceived ease of use.

Peer influence, as noted by Rouidi et al. had the least impact on attitudes toward the use of e‐prescribing [24]. This indicates that social influence may not play a significant role in the acceptance of e‐prescribing among Iranian physicians and pharmacists. Therefore, it is important to explore the variable of “social influence”, which currently lacks sufficient support and relevance [24]. Physicians may be resistant to adopting electronic prescriptions, often due to their established reliance on traditional methods or concerns about the potential consequences of implementing new technologies.

Technology self‐efficacy had a positive and significant effect on attitudes toward technology. By enhancing self‐efficacy, we not only shape behavioral intentions but also encourage positive actions related to technology. Previous research on technology acceptance and adoption has consistently shown that self‐efficacy is a crucial predictor of attitudes toward technology [52, 53, 54]. Increased confidence in technological abilities leads to greater willingness to adopt e‐prescription systems [52]. However, this finding contrasts with the work of Rati and Kemeny, who suggested that technological readiness does not necessarily lead to trust or intention to use technology. Their research also indicates that technological readiness might negatively affect the intention to use technology [55]. This discrepancy emphasizes the multifaceted nature of technology adoption and underscores the importance of conducting further research to identify the factors that affect technology acceptance.

The results of our hypothesis testing revealed that the perceived usefulness of the electronic prescription system does not significantly influence users' attitudes toward its use in our model. This indicates that an individual's attitude toward use cannot solely be formed from their perception of usefulness. These findings are consistent with Wimbo Raksadigiri and Wahyuni's study, which also concluded that perceived usefulness does not have a significant effect on attitudes toward use [47]. However, these findings were contradicted by some of the studies' findings. Perceived ease of use affects perceived usefulness, attitude toward use, and its impact on behavioral intention to use [54, 55]. This suggests that other factors beyond perceived usefulness play a significant role in shaping user attitudes. Understanding these relationships is crucial for enhancing user engagement and system adoption.

Perceived ease of use does not significantly affect users' attitudes toward utilizing the system. These findings contrast with prior research that demonstrated a substantial impact of perceived ease of use on attitudes toward use [47, 54, 56]. Such discrepancies may arise from factors including user demographics, the complexity of technology, or varying organizational cultures that influence how users interact with the system. Understanding these factors may offer a deeper insight into user attitudes.

Trust is generally considered essential, yet it did not significantly influence users' intention to use technology in our study. This finding contrasts with the results of earlier research, which posits that trust plays a pivotal role in technology utilization [55]. Trust enables users to feel confident that they can achieve their goals using technology. However, it is essential to consider other influencing factors. Variations in system quality and cultural aspects may contribute to differences in user experiences. Additionally, organizational factors such as physicians' heavy workloads, limited time and resources, and inadequate support from management can significantly impact the validity of these assumptions. Vance et al. emphasize that both system quality and cultural aspects significantly contribute to the development of trust in information technology systems [57]. Recognizing these elements can enhance user engagement and the overall effectiveness of technology.

Finally, contrary to expectations, a positive attitude did not significantly influence the behavioral intention to use technology. These results are inconsistent with findings from other studies, which have shown that positive attitudes do have a significant positive effect on the behavioral intention to adopt technology [47, 55, 56, 58]. This may be due to physicians' busy schedules and limited experience with information systems. Studies indicate that inadequate technical infrastructure—including internet speed, hardware, and software equipment—as well as insufficient training on the use of electronic prescriptions, are significant challenges facing electronic prescriptions in Iran [31, 32, 33, 59]. These infrastructure issues may contribute to the rejection of the hypothesis.

### 4.1 Potential Strengths, Limitations, and Suggestions for Future Research

This study significantly advances our understanding of e‐prescription systems. By integrating the TAM and the UTAUT, our study provided a strong theoretical framework to explain the myriad factors governing the adoption of electronic prescription systems. The use of MPLUS for statistical analysis strengthened our methodology and increased flexibility and accuracy for modeling latent variables–covariates.

However, it is important to note that the cross‐sectional design and regional focus may limit the generalizability of the findings. To enhance future research, it is recommended to include diverse samples and investigate potential moderating factors. Adopting objective data collection methods could significantly improve the validity of the results. Qualitative studies that delve into user experiences also contribute valuable insights into the adoption process. Finally, it is advisable to explore the reasons behind the nonsignificant findings related to the relationships between trust, attitude, self‐efficacy, and the constructs of perceived usefulness and ease of use. Future research could expand the scope to include moderating variables identified in the literature, thereby gaining a deeper understanding of the factors influencing e‐prescribing adoption. For this purpose, qualitative research methods such as in‐depth interviews with physicians and content analysis of their opinions can be used. Also, examining the role of intervening factors such as education, organizational support, and user experience can help to understand this issue better.

## 5 Conclusion

This study explored the factors that influence the adoption of e‐prescribing among healthcare providers in Iran, where e‐prescribing has been mandatory since December 2021. By combining the TAM with the UTAUT, this study provides a comprehensive understanding of e‐prescribing acceptance within the Iranian healthcare system. Our findings reveal several key determinants that significantly impact the uptake of e‐prescribing among healthcare professionals in Iran. Specifically, we discovered that perceived ease of use positively affects perceived usefulness, which, in turn, significantly influences trust in the system. Additionally, technology self‐efficacy plays a crucial role in shaping positive attitudes toward e‐prescribing. While our study confirmed the importance of these factors, it also revealed some unexpected results. Notably, perceived usefulness did not have a direct effect on attitudes toward use, and trust did not significantly impact the intention to use the system. These findings highlight the complex interplay of factors influencing e‐prescribing adoption and suggest that other contextual or cultural variables may be influencing this phenomenon in Iran. Furthermore, the lack of significant influence from peer pressure indicates that social influence may not be a primary driver of adoption in this context, which calls for further investigation. The insights gained from this study will be invaluable for policymakers, healthcare administrators, and technology developers dedicated to promoting the successful integration of e‐prescribing systems.

## Author Contributions

**Mahdie ShojaeiBaghini:** conceptualization, funding acquisition, investigation, writing – original draft, writing – review and editing, visualization, validation, software, formal analysis, project administration, resources, supervision, data curation, and methodology.

## Ethics Statement

This project was approved by the Ethics Committee of Kerman University of Medical Sciences (IR.KMU.REC.1401.504). The author is accountable for all aspects of the work and ensures that questions related to the accuracy or integrity of any part of the work are appropriately investigated and resolved.

## Consent

The purpose of this study was explained to all participants and their contribution was voluntary. Oral informed consent was received from all participants.

## Conflicts of Interest

The author declares no conflicts of interest.

## Transparency Statement

The author Mahdie ShojaeiBaghini affirms that this manuscript is an honest, accurate, and transparent account of the study being reported; that no important aspects of the study have been omitted; and that any discrepancies from the study as planned (and, if relevant, registered) have been explained.

## Data Availability

The data set presented in the study is available on request from the corresponding author during submission or after publication.
